# Supplementary material for: Serum-Based Proteomics Profiling in Adult Patients with Cystic Fibrosis
Source: Int J Mol Sci. 2020 Oct 8;21(19):7415. doi: 10.3390/ijms21197415 (PMC7582405; doi:10.3390/ijms21197415)
Supplement: Supplementary file 1 [file ijms-21-07415-s001.pdf]

Supplementary Materials for:

Serum-based Proteomics Profiling in Adult Patients with Cystic Fibrosis

Hicham Benabdelkamel[1], Hanadi Alamri[2], Meshail Okla[3], Afshan Masood[1], Mai Abdel Jabar[4], Ibrahim O. Alanazi[5], Assim A. Alfadda[1,6], Imran Nizami[7], Majed Dasouki[4], Anas M. Abdel Rahman[2,4,8*]

[1] Proteomics Resource Unit, Obesity Research Center, College of Medicine, King Saud University, PO. Box 2925 (98), Riyadh 11461, Kingdom of Saudi Arabia

[2] Department of Biochemistry and Molecular Medicine,College of Medicine, Al Faisal University, Riyadh, Kingdom of Saudi Arabia.

[3] Department of Community Health Sciences, College of Applied Medical Sciences, King Saud University, 183T11, Riyadh 11495, Kingdom of Saudi Arabia.

[4] Department of Genetics, King Faisal Specialist Hospital and Research Centre (KFSHRC), Zahrawi Street, Al Maather, Riyadh 11211, Kingdom of Saudi Arabia.

[5] The National Center for Biotechnology (NCB), Life Science and Environment Research Institute, King Abdulaziz City for Science and Technology (KACST), PO. Box 6086, Riyadh, Kingdom of Saudi Arabia.

[6] Department of Medicine, College of Medicine, King Saud University, PO. Box 2925 (98), Riyadh 11461, Kingdom of Saudi Arabia.

[7] Lung Transplant Section, Organ Transplant Center, King Faisal Specialist Hospital and Research Center, Zahrawi Street, Al Maather, Riyadh 11211, Saudi Arabia.

[8] Department of Chemistry, Memorial University of Newfoundland, St. John's, NL, A1B 3X7, Canada.

*Correspondence: aabdelrahman46@kfshrc.edu.sa; Tel: +966114647272 Ext. 36481(A.A.R); madasouki@kfshrc.edu.sa; Tel: +966114647272 Ext. 24081 (M.D.)

Figure S1. (A) Representative image of statistically significant differentially expressed (ANOVA $p \leq 0.05$ and fold-change ≥ 1.5) protein spots from the serum samples with red color depicting the upregulated proteins and blue color depicting the down regulated proteins in CF samples compared to control samples (B) Representative image of CF gels with top 20 up-regulated proteins in CF vs control group.

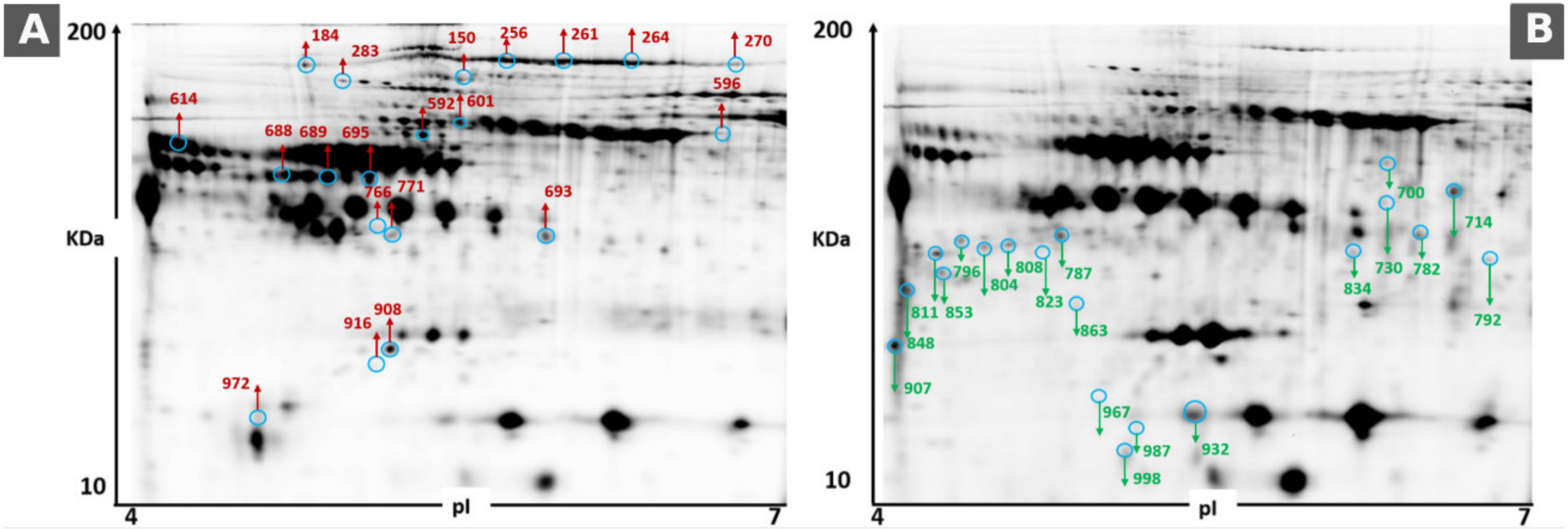

Figure S2. Representative extracted ion chromatograms for the selected proteins' signature peptides based on the multiple-reaction monitoring method developed for validation.

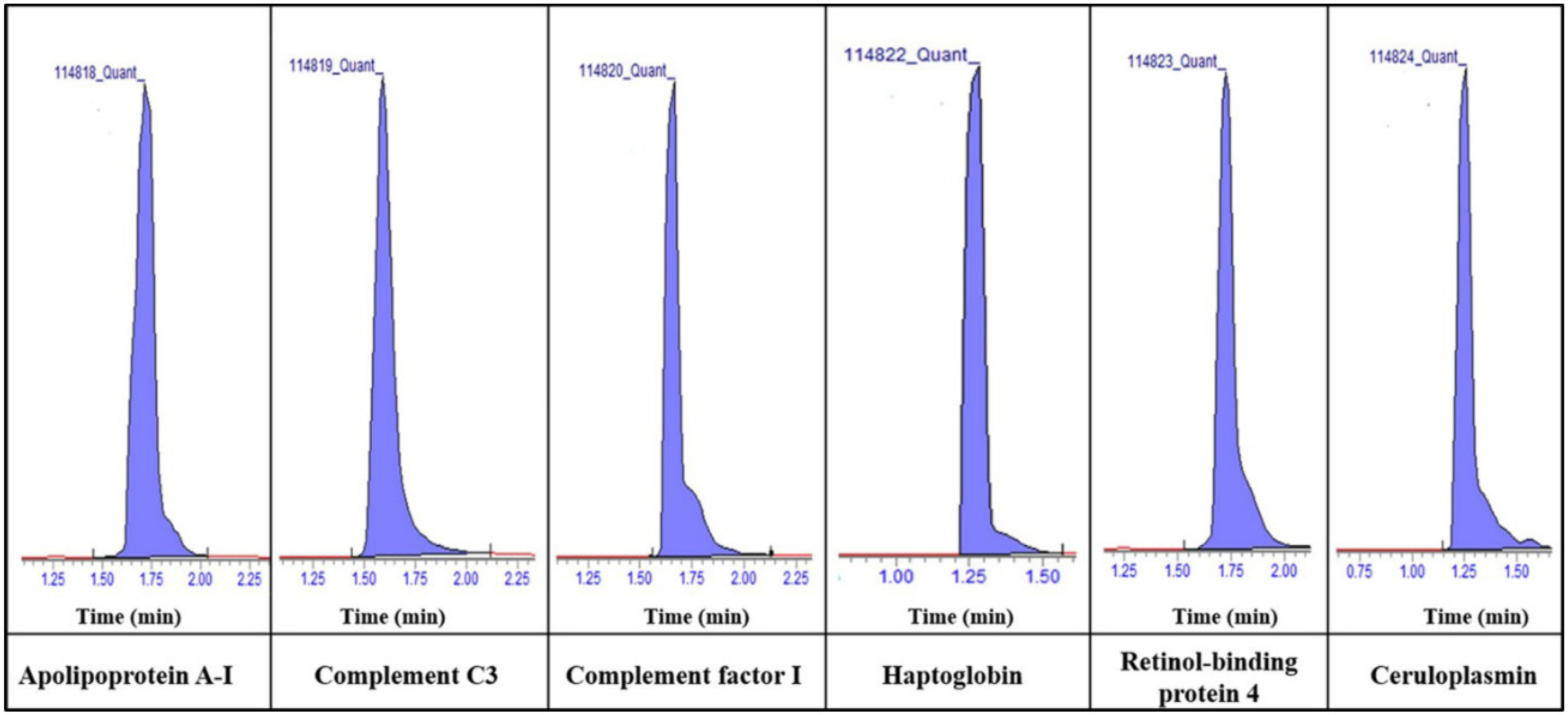

Figure S3. PCA plot of the first two principal components. Together, they explained 67% of the selected spot's variability. The colored dots and numbers represent the gels and spots, respectively.

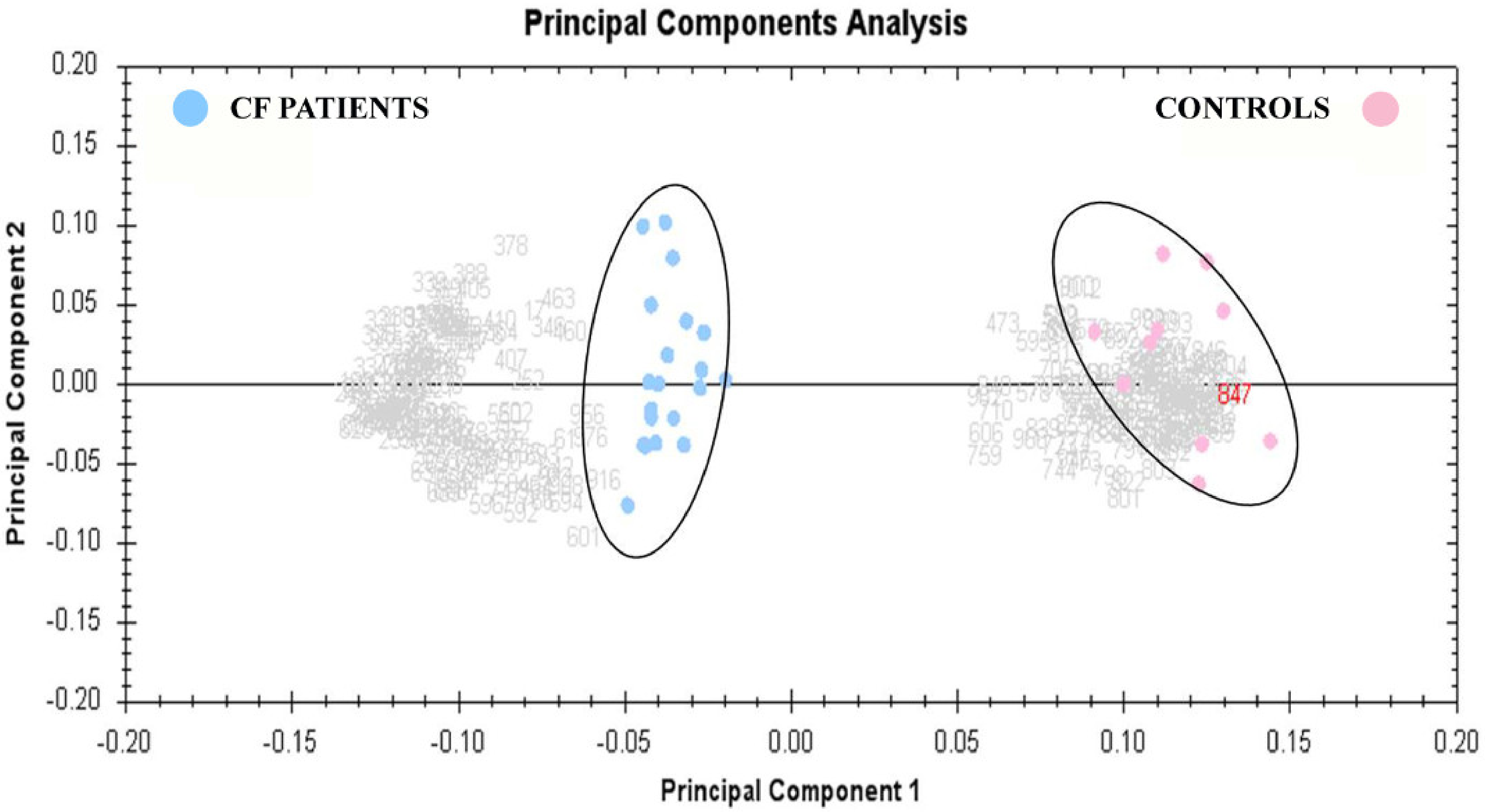

Figure S4. Expression profiles, separated into clusters of expression patterns, indicating the number of spots for each cluster. Each line represents the standardized abundance of a spot across all gels and belongs to one of the clusters generated by hierarchical cluster analysis. The spots with increased abundance indicate the 80 proteins up-regulated in the CF group (Figure S 3B). The spots with decreased abundance indicate the 54 proteins down-regulated in the CF group (Figure S3A) (Progenesis Same Spots).

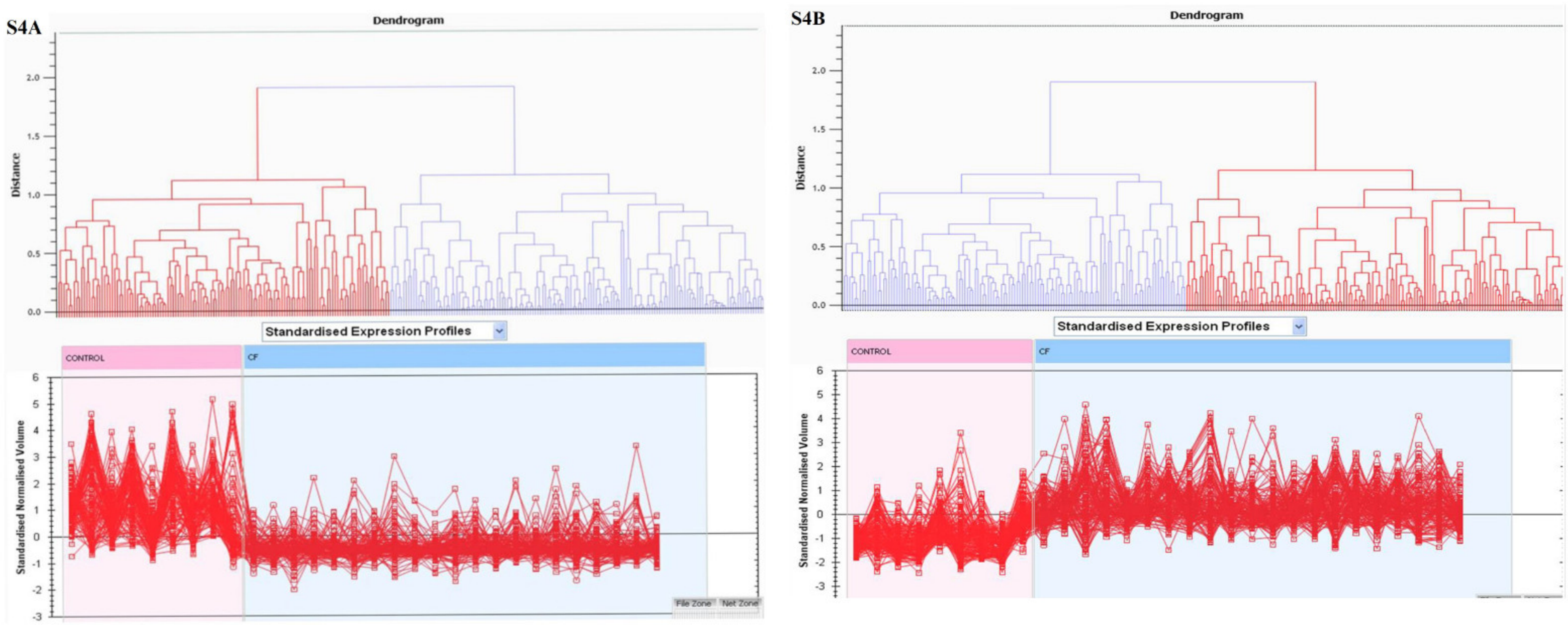

Figure S5. Pathways and canonical pathways identified in the functional IPA.

Top Networks

ID	Associated Network Functions	Score
1	Metabolic Disease, Neurological Disease, Organismal Injury and Abnormalities	33
2	Cellular Function and Maintenance, Cardiovascular Disease, Hereditary Disorder	18
3	Cancer, Gastrointestinal Disease, Organismal Injury and Abnormalities	18

Top Canonical Pathways

Name	p-value	Overlap
FXR/RXR Activation	2.12E-14	8.8 % 11/125
LXR/RXR Activation	6.79E-13	8.3 % 10/121
Acute Phase Response Signaling	2.76E-11	5.7 % 10/175
IL-12 Signaling and Production in Macrophages	1.09E-07	4.8 % 7/146
Production of Nitric Oxide and Reactive Oxygen Species in Macrophages	7.77E-07	3.6 % 7/195

Figure S6. Comparative depiction of the differentially abundant proteins categorized into groups according to their molecular function (A), biological process (B), and cellular component (C) using the PANTHER (Protein ANalysis THrough Evolutionary Relationships) classification system (http://www.pantherdb.org/). The representative pie chart shows the percentage of identified proteins involved in each of the different functional categories.

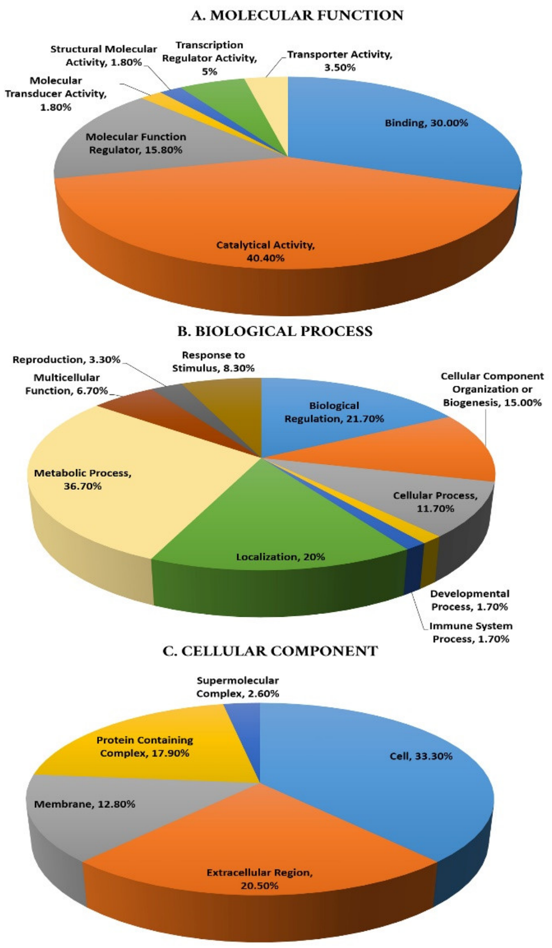

Figure S7. Power calculation for determining the minimum number of required biological variants for the 2-DIGE analysis. The power curve was used to calculate the sample size required to find a significant difference with a fold-change of ≥1.5 between two paired groups, with 99.3% of the data and>80% power.

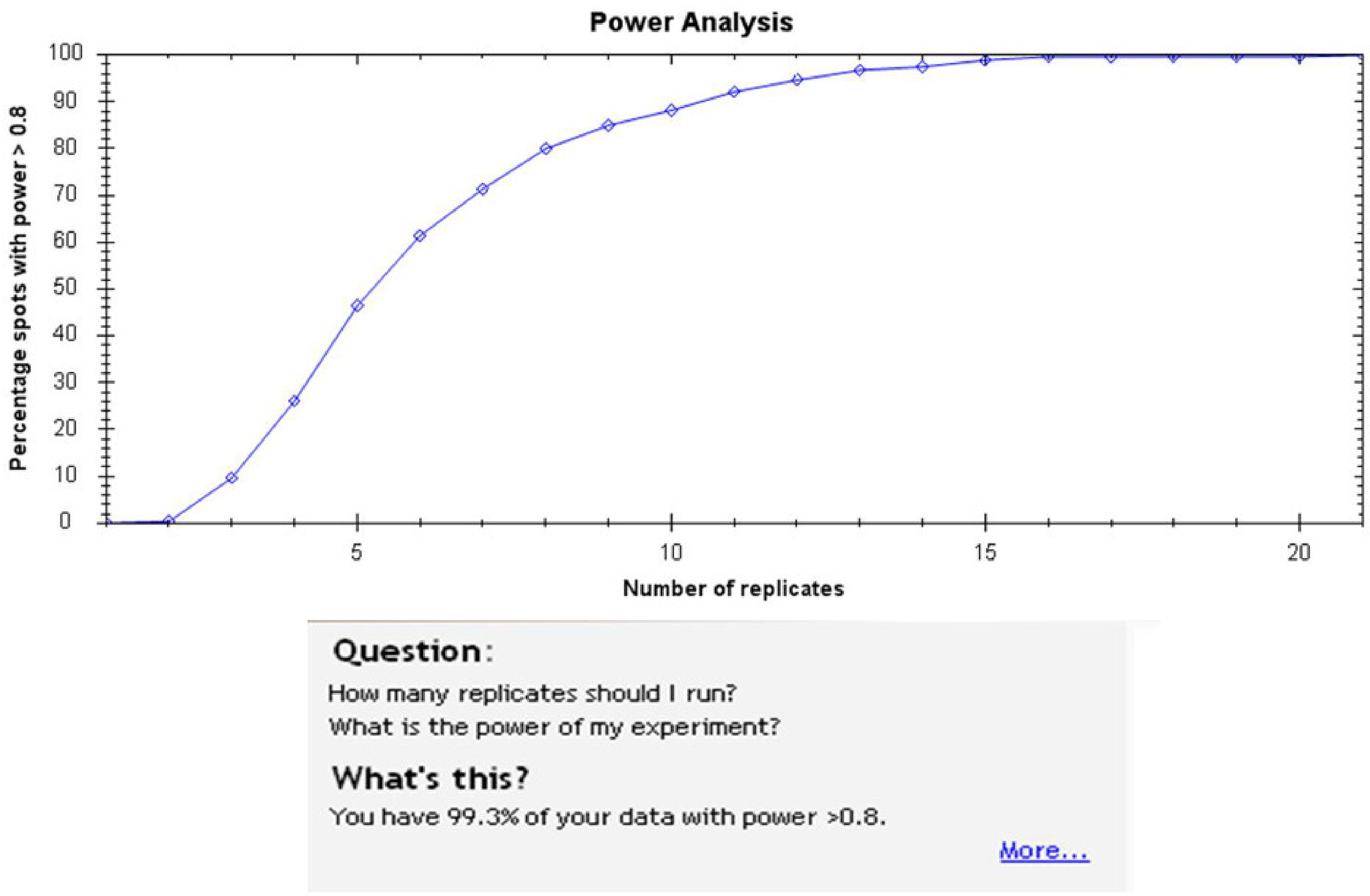

Table S1. Demographics and routine clinical data of the CF patients participated in this study.

Patient #	Age (years)	Gender	Total serm vitamin D	Serum vitamin E	BMI	CFRD	IgE	Pancreatic insufficiency
**CF1**	23	M	37	7.3	29	No	37.4	Yes
**CF2**	22	F	10.8	4.4	18	No	2.5	Yes
**CF3**	19	F	39	12.5	19	Yes	1176	Yes
**CF4**	20	M	34.8	5.1	17.4	No	496	Yes
**CF5**	22	F	58	13.4	19	No	103	Yes
**CF6**	17	F	73.8	5.1	15.9	Yes	25.3	Yes
**CF7**	22	M	61.7	9.8	15	Yes	143	Yes
**CF9**	18	F	43.9	6.2	20	No	34	Yes
**CF10**	28	M	62.9	7.7	14	No	364	Yes
**CF11**	26	F	80	20.9	15.1	No	ND	Yes
**CF12**	34	F	42.9	12.7	20	No	99.2	Yes
**CF13**	20	M	51.7	19	14	No	67.7	Yes
**CF14**	17	M	46.9	7.9	21	No	310	Yes
**CF16**	18	F	78.8	23	20	No	113	Yes
**CF22**	14	F	20.9	12.2	15	No	47.1	Yes
**CF24**	17	F	22.6	6.5	26	No	31.7	Yes
**CF25**	20	F	33.5	ND	23	No	ND	Yes
**CF26**	26	M	63.4	14.3	16	No	5	Yes
**CF27**	14	M	80.8	10.1	15	No	111	Yes
**CF29**	26	F	53.9	ND	18	No	ND	Yes
**CF30**	12	M	119	20.2	19.5	No	386	Yes
**CF31**	24	F	99	27.8	21.2	No	26.1	Yes
**CF32**	20	F	57	10.6	17.2	No	96.1	Yes
**CF33**	22	F	92	13.4	18.2	No	2399	No
**CF34**	18	M	49.7	8.9	15	No	891	Yes

**CF40**	18	F	48.3	9.5	15	Yes	23.3	Yes
**CF41**	14	M	45.8	7.8	17.6	Yes	20.4	Yes
**CF42**	18	M	59	7.9	20	No	51.5	Yes

BMI body mass index, CFRD cystic fibrosis relared diabetes mellitus, serum IgE ref. reange: 5-500 KU/L), serum total vitamin D ref. range: 13-76 nmol/L, serum vitamin E reference range: 5.5-15.5 mg/L.

Table S2. Mass spectrometry list of significant differentially abundant proteins between the controls and CF patients identified in samples using 2D-DIGE. The protein name, accession number, Mascot score, MS % coverage, protein MW, and pI values, according to the Uniprot database, are listed.

Spot No	Accession No	Protein Name	MASCOT ID	Pi	MW	Cov %	Score
804	P32019	Type II inositol 1,4,5-trisphosphat	I5P2_HUMAN	5.377	114035	19	62
836	P32019	Type II inositol 1,4,5-trisphosphat	I5P2_HUMAN	5.377	114035	25	60
792	O95932	Protein-glutamine gamma-glutamyltransferase 6	TGM3L _HUMAN	6.85	80061	23	56
853	Q8TDY2	RB1-inducible coiled-coil protein 1	RBCC1_HUMAN	5.3	185085	21	72
796	Q8IZP2	Putative protein FAM10A4	F10A4	5.01	27561	26	56
787	O75923	Dysferlin	DYSF_HUMAN	5.44	239254	14	54
184	P01023	Alpha-2-macroglobulin	A2MG_HUMAN	6.0	164614	29	89
823	P06858	Lipoprotein lipase	LIPL_HUMAN	8.37	53699	25	59
808	Q2M2D7	TBC1 domain family member 28	TBC28_HUMAN	9.7	24285	36	55
932	Q8TDE3	Ribonuclease 8	RNAS8_HUMAN	8.69	17600	70	52
811	P32019	Type II inositol 1,4,5-trisphosphat	I5P2_HUMAN	5.37	114035	19	56
163	P01023	Alpha-2-macroglobulin	A2MG_HUMAN	6.0	164614	18	55
635	P01042	Kininogen-1	KNG1_HUMAN	6.34	72996	23	58
243	P01023	Alpha-2-macroglobulin	A2MG_HUMAN	6.0	164614	19	75
247	P01023	Alpha-2-macroglobulin	A2MG_HUMAN	6.0	164614	21	87
916	P01042	Kininogen-1	KNG1_HUMAN	6.34	72996	23	58
614	P01011	Alpha-1-antichymotrypsin	AACT_HUMAN	5.33	47792	31	81
261	P01023	Alpha-2-macroglobulin	A2MG_HUMAN	6.0	164614	36	178
264	P01023	Alpha-2-macroglobulin	A2MG_HUMAN	6.0	164614	36	187
856	Q49AM3	Tetratricopeptide repeat protein 31	TTC31_HUMAN	8.52	57753	22	49
848	Q96LK0	Centrosomal protein of 19 kDa	CC034_HUMAN	5.24	19382	28	56
998	Q9UL68	Myelin transcription factor 1-like	MYT1L	4.87	135182	11	75
260	P01023	Alpha-2-macroglobulin	A2MG_HUMAN	6.0	164614	31	110
987	Q86WV5	CST complex subunit TEN1	YQ023_HUMAN	7.77	13887	89	56
273	P01023	Alpha-2-macroglobulin	A2MG_HUMAN	6.0	164614	31	105
283	P01023	Alpha-2-macroglobulin	A2MG_HUMAN	6.0	164614	30	101
276	P01023	Alpha-2-macroglobulin	A2MG_HUMAN	6.0	164614	34	154
834	O60869	Endothelial differentiation-related factor 1	EDF1_HUMAN	9.95	16359	29	49
225	P01023	Alpha-2-macroglobulin	A2MG_HUMAN	6.0	164614	33	182
857	Q9UL68	Myelin transcription factor 1-like protein	MYT1L_HUMAN	4.87	135182	22	54
256	P01023	Alpha-2-macroglobulin	A2MG_HUMAN	6.0	164614	23	65

259	P01023	Alpha-2-macroglobulin	A2MG_HUMAN	6.0	164614	37	129
149	P01023	Alpha-2-macroglobulin	A2MG_HUMAN	6.0	164614	19	47
234	P01023	Alpha-2-macroglobulin	A2MG_HUMAN	6.0	164614	21	69
789	Q99570	Phosphoinositide 3-kinase regulatory subunit 4	PI3R4__HUMAN	6.74	154318	11	54
150	Q9H2F9	Coiled-coil domain-containing protein 68	CCD68__HUMAN	8.78	39073	22	56
893	P02647	Apolipoprotein A-I	APOA1_HUMAN	5.56	30759	62	176
277	P01023	Alpha-2-macroglobulin	A2MG_HUMAN	6.0	164614	33	129
294	P01023	Alpha-2-macroglobulin	A2MG_HUMAN	6.0	164614	30	113
610	P01011	Alpha-1-antichymotrypsin	AACT_HUMAN	5.33	47792	34	68
289	P01023	Alpha-2-macroglobulin	A2MG_HUMAN	6.0	164614	23	68
863	Q6UWF9	Protein FAM180A	F180A_HUMAN	8.59	19834	36	54
689	P01009	Alpha-1-antitrypsin	A1AT_HUMAN	5.37	46878	36	104
285	P01023	Alpha-2-macroglobulin	A2MG_HUMAN	6.0	164614	22	82
291	P01023	Alpha-2-macroglobulin	A2MG_HUMAN	6.0	164614	36	129
281	P01023	Alpha-2-macroglobulin	A2MG_HUMAN	6.0	164614	27	114
695	P01009	Alpha-1-antitrypsin	A1AT_HUMAN	5.37	46878	27	55
87	P01023	Alpha-2-macroglobulin	A2MG_HUMAN	6.0	164614	24	77
907	P02647	Apolipoprotein A-I	APOA1_HUMAN	5.56	30759	46	98
567	Q96PX9	Pleckstrin	PKH4B_HUMAN	6.22	141345	21	65
253	P01023	Alpha-2-macroglobulin	A2MG_HUMAN	6.0	164614	31	122
334	P07911	Uromodulin	UROM_HUMAN	5.05	72451	38	94
270	P08603	Complement factor H	CFAH_HUMAN	6.21	143680	28	71
326	P00450	Ceruloplasmin	CERU_HUMAN	5.44	122983	35	148
688	P01009	Alpha-1-antitrypsin	A1AT_HUMAN	5.37	46878	29	76
331	P00450	Ceruloplasmin	CERU_HUMAN	5.44	122983	28	143
638	P01042	Kininogen-1	KNG1_HUMAN	6.34	72996	25	65
255	P01023	Alpha-2-macroglobulin	A2MG_HUMAN	6.0	164614	28	100
570	P02768	Serum albumin	ALBU_HUMAN	5.92	71317	31	69
771	P01024	Complement C3	CO3_HUMAN	6.02	188569	16	49
967	Q9UL68	Myelin transcription factor 1-like protein	MYT1L_HUMAN	4.87	135182	11	73
656	P01009	Alpha-1-antitrypsin	A1AT_HUMAN	5.37	46878	38	92
730	Q6JEL2	Kelch-like protein 10	KLH10_HUMAN	5.47	69923	25	49
659	P01009	Alpha-1-antitrypsin	A1AT_HUMAN	5.37	46878	47	96
651	P01009	Alpha-1-antitrypsin	A1AT_HUMAN	5.37	46878	53	135
338	P00450	Ceruloplasmin	CERU_HUMAN	5.44	122983	19	62

714	Q96QP1	Alpha-protein kinase 1	ALPK1_HUMAN	5.81	140210	18	59
785	P02774	Vitamin D-binding protein, DBP	VTDB_HUMAN	5.4	54526	50	100
362	P01023	Alpha-2-macroglobulin	A2MG_HUMAN	6.0	164614	29	107
596	P00450	Ceruloplasmin	CERU_HUMAN	5.44	122983	28	81
649	P01009	Alpha-1-antitrypsin	A1AT_HUMAN	5.37	46878	38	95
257	P01023	Alpha-2-macroglobulin	A2MG_HUMAN	6.0	164614	20	77
591	O00487	26S proteasome non-ATPase regulatory subunit 14	PSDE_HUMAN	6.06	34726	45	54
782	P00738	Haptoglobin	HPT_HUMAN	6.13	45861	43	73
266	P01023	Alpha-2-macroglobulin	A2MG_HUMAN	6.0	164614	27	80
763	P62906	60S ribosomal protein L10a	RL10A_HUMAN	9.94	24987	35	51
263	P01023	Alpha-2-macroglobulin	A2MG_HUMAN	6.0	164614	32	111
919	P02753	Retinol-binding protein 4	RET4_HUMAN	5.76	23337	61	66
589	P02768	Serum albumin	ALBU_HUMAN	5.92	71317	35	68
367	P00450	Ceruloplasmin	CERU_HUMAN	5.4	122983	24	75
801	Q96SI1	BTB/POZ domain-containing protein KCTD15	KCD15_HUMAN	7.05	32264	43	66
339	P00450	Ceruloplasmin	CERU_HUMAN	5.4	122983	21	56
829	P24310	Cytochrome c oxidase subunit 7A1	CY7A1_HUMAN	10.2	9169	51	52
709	Q8TDE3	Ribonuclease 8	RNAS8_HUMAN	8.69	17600	70	54
332	Q14833	Metabotropic glutamate receptor 4	GRM4_HUMAN	9.07	103285	26	55
645	P01009	Alpha-1-antitrypsin	A1AT_HUMAN	5.37	46878	34	69
668	Q9BQ50	Three prime repair exonuclease 2	TREX2_HUMAN	6.37	31172	27	49
408	P43652	Afamin	AFAM_HUMAN	5.64	70963	28	51
592	P15622	Zinc finger protein 250	ZN250_HUMAN	8.65	65252	20	48
972	Q9NR11	Zinc finger protein 302	ZN302_HUMAN	9.1	56033	43	65
650	P01009	Alpha-1-antitrypsin	A1AT_HUMAN	5.37	46878	25	64
405	P00450	Ceruloplasmin	CERU_HUMAN	5.44	122983	37	98
345	P00450	Ceruloplasmin	CERU_HUMAN	5.44	122983	35	79
389	P01023	Alpha-2-macroglobulin	A2MG_HUMAN	6.0	164614	22	64
700	P01877	Immunoglobulin heavy constant alpha 2	IGHA2_HUMAN	5.71	37301	30	70
773	P05156	Complement factor I	CFAI_HUMAN	7.72	68071	17	64
900	P02647	Apolipoprotein A-I	APOA1_HUMAN	5.56	30759	61	156
820	P02647	Apolipoprotein A-I	APOA1_HUMAN	5.56	30759	47	85
343	P00450	Ceruloplasmin	CERU_HUMAN	5.44	122983	26	63
908	Q9NT22	EMILIN-3	EMIL3	7.84	83622	22	50

693	P01009	Alpha-1-antitrypsin	A1AT_HUMAN	5.37	46878	44	61
896	P02647	Apolipoprotein A-I	APOA1_HUMAN	5.56	30759	58	161
374	P01023	Alpha-2-macroglobulin	A2MG_HUMAN	6.0	164614	18	73
388	Q14833	Metabotropic glutamate receptor 4, mGluR4	GRM4_HUMAN	9.07	103285	24	52
473	P24666	Low molecular weight phosphotyrosine protein phosphatase	PPAC_HUMAN	6.30	18487	53	53
1012	Q6ZN57	Zinc finger protein 2 homolog, Zfp-2	ZFP2_HUMAN	8.91	54360	39	51
502	P04217	Alpha-1B-glycoprotein	A1BG_HUMAN	5.58	54809	37	92
611	Q9H0J9	Poly [ADP-ribose] polymerase 12	PAR12_HUMAN	8.84	80496	28	66
601	P00450	Ceruloplasmin	CERU_HUMAN	5.44	122983	23	76
558	Q9NXU5	ADP-ribosylation factor-like protein 15	ARL15_HUMAN	5.41	23261	48	57
641	P01009	Alpha-1-antitrypsin	A1AT_HUMAN	5.37	46878	30	54
839	P31751	RAC-beta serine/threonine-protein kinase	AKT2_HUMAN	5.98	56132	33	53
376	P00450	Ceruloplasmin	CERU_HUMAN	5.44	122983	23	87
840	O95922	Probable tubulin polyglutamylase TTLL1	TTLL1_HUMAN	8.87	49356	31	60
766	Q6UXP9	Putative uncharacterized protein	Y0001	9.67	20049	35	49
759	P00738	Haptoglobin	HPT_HUMAN	6.13	45861	37	70
966	P00738	Haptoglobin	HPT_HUMAN	6.13	45861	39	71
377	P01023	Alpha-2-macroglobulin	A2MG_HUMAN	6.0	164614	20	63
351	Q8NDZ2	SUMO-interacting motif-containing protein 1	CE025_HUMAN	6.2	97919	18	60
463	Q8TCP9	Protein FAM200A	CG038_HUMAN	5.82	66918	22	65
642	P01009	Alpha-1-antitrypsin	A1AT_HUMAN	5.37	46878	51	182
560	P04217	Alpha-1B-glycoprotein	A1BG_HUMAN	5.58	54809	40	107
559	P00450	Ceruloplasmin	CERU_HUMAN	5.44	122983	22	81
460	P00450	Ceruloplasmin	CERU_HUMAN	5.44	122983	15	71
710	P01008	Antithrombin-III	ANT3_HUMAN	6.32	53025	29	64
982	Q8TAA9	Vang-like protein 1	VANG1_HUMAN	8.94	60222	16	51
949	Q8TDE3	Ribonuclease 8	RNAS8	8.69	17600	66	54
604	P02790	Hemopexin	HEMO_HUMAN	5044	52385	31	63
465	P04217	Alpha-1B-glycoprotein	A1BG_HUMAN	5.58	54809	32	62
404	P00450	Ceruloplasmin	CERU_HUMAN	5.44	122983	35	110
449	P02768	Serum albumin	ALBU_HUMAN	5.92	71317	37	100
895	P02647	Apolipoprotein A-I,	APOA1_HUMAN	5.56	30759	46	97
598	P02790	Hemopexin	HEMO_HUMAN	6.51	52385	54	109
862	P24310	Cytochrome c oxidase subunit 7A1	CX7A1_HUMAN	10.12	9169	72	58

Table S3. The potential biomarker proteins based on the ROC curve analysis using the PLS-DA model.

Spot ID	Accession No	Protein Name	Volcano Plot Analysis				ROC Analysis			
			FC	log2(FC)	FDRp	log10(p)	AUC	Pval	FC	clusters
804	P32019	Type II inositol 1,4,5-trisphosphat	0.066266	-3.9156	6.36E-09	8.1964	0.968254	4.75E-11	-3.32185	2
792	O95932	Protein-glutamine gamma-glutamyltransferase 6	0.11217	-3.1562	5.35E-08	7.272	0.962963	7.98E-10	-2.54495	2
808	Q2M2D7	TBC1 domain family member 28	0.12173	-3.0382	9.95E-08	7.0023	0.962963	2.23E-09	-2.62786	2
811	P32019	Type II inositol 1,4,5-trisphosphat	0.082225	-3.6043	4.86E-07	6.3137	0.957672	2.02E-08	-2.60417	2
932	Q8TDE3	Ribonuclease 8	0.11692	-3.0964	4.86E-07	6.3137	0.957672	2.02E-08	-2.60417	2
834	O60869	Endothelial differentiation-related factor 1	0.24606	-2.0229	4.86E-07	6.3137	0.936508	2.17E-08	-1.55997	2
796	Q8IZP2	Putative protein FAM10A4	0.09981	-3.3247	6.74E-07	6.1713	0.910053	3.52E-08	-2.75489	2
787	O75923	Dysferlin	0.12727	-2.9741	7.62E-07	6.1183	0.915344	4.55E-08	-2.30206	2
836	P32019	Type II inositol 1,4,5-trisphosphat	0.05959	-4.0688	7.64E-07	6.117	0.910053	5.13E-08	-3.33513	2
998	Q9UL68	Myelin transcription factor 1-like	0.070859	-3.8189	1.24E-06	5.9051	0.968254	9.28E-08	-3.15813	2
853	Q8TDY2	RB1-inducible coiled-coil protein 1	0.094443	-3.4044	1.33E-06	5.8767	0.920635	1.09E-07	-2.79633	2
261	P01023	Alpha-2-macroglobulin	3.5034	1.8087	1.41E-06	5.8499	0.94709	1.27E-07	2.408687	1
264	P01023	Alpha-2-macroglobulin	2.934	1.5529	2.31E-06	5.6362	0.941799	2.24E-07	2.198797	1
243	P01023	Alpha-2-macroglobulin	2.4216	1.276	3.20E-06	5.4949	0.904762	3.58E-07	1.887451	4
848	Q96LK0	Centrosomal protein of 19 kDa	0.11776	-3.0861	4.74E-06	5.3241	0.904762	7.26E-07	-2.45095	2

907	P02647	Apolipoprotein A-I	0.22194	-2.1717	4.74E-06	5.3241	0.94709	7.54E-07	-1.82351	5
789	Q99570	Phosphoinositide 3-kinase regulatory subunit 4	0.29364	-1.7679	4.74E-06	5.3241	0.941799	7.78E-07	-1.20642	2
856	Q49AM3	Tetratricopeptide repeat protein 31	0.30516	-1.7124	4.74E-06	5.3241	0.931217	7.54E-07	-1.28322	2
184	P01023	Alpha-2-macroglobulin	2.3736	1.2471	4.74E-06	5.3241	0.89418	7.19E-07	1.789971	4
614	P01011	Alpha-1-antichymotrypsin	2.2856	1.1926	4.74E-06	5.3241	0.957672	7.75E-07	1.549498	4
247	P01023	Alpha-2-macroglobulin	2.0344	1.0246	5.66E-06	5.2469	0.925926	9.72E-07	1.530206	4
857	Q9UL68	Myelin transcription factor 1-like protein	0.27348	-1.8705	7.19E-06	5.1435	0.941799	1.29E-06	-1.42493	2
801	Q96SI1	BTB/POZ domain-containing protein KCTD15	0.41014	-1.2858	1.42E-05	4.8473	0.920635	2.75E-06	-0.8172	2
260	P01023	Alpha-2-macroglobulin	2.2671	1.1809	1.42E-05	4.8473	0.910053	2.76E-06	1.764769	1
256	P01023	Alpha-2-macroglobulin	2.9589	1.565	1.57E-05	4.8029	0.936508	3.17E-06	2.218563	1
823	P06858	Lipoprotein lipase	0.28567	-1.8076	2.02E-05	4.6955	0.936508	4.36E-06	-1.49639	2
259	P01023	Alpha-2-macroglobulin	2.17	1.1177	2.02E-05	4.6955	0.920635	4.33E-06	1.65228	1
987	Q86WV5	CST complex subunit TEN1	0.10586	-3.2397	2.18E-05	4.6607	0.904762	4.89E-06	-2.76194	2
730	Q6JEL2	Kelch-like protein 10	0.27101	-1.8836	3.50E-05	4.4558	0.920635	8.10E-06	-1.50371	5
863	Q6UWF9	Protein FAM180A	0.19199	-2.3809	4.86E-05	4.3138	0.867725	1.20E-05	-1.72259	2
919	P02753	Retinol-binding protein 4	0.33975	-1.5575	4.97E-05	4.3033	0.925926	1.30E-05	-1.20139	5
273	P01023	Alpha-2-macroglobulin	2.1147	1.0804	4.97E-05	4.3033	0.89418	1.29E-05	1.651603	1
225	P01023	Alpha-2-macroglobulin	2.132	1.0922	5.11E-05	4.2918	0.888889	1.41E-05	1.743094	1

785	P02774	Vitamin D-binding protein, DBP	0.28841	-1.7938	5.12E-05	4.291	0.915344	1.45E-05	-1.36889	2
234	P01023	Alpha-2-macroglobulin	2.2806	1.1894	5.31E-05	4.2749	0.915344	1.55E-05	1.858335	1
709	Q8TDE3	Ribonuclease 8	0.44246	-1.1764	5.76E-05	4.2393	0.931217	1.76E-05	-0.78339	5
967	Q9UL68	Myelin transcription factor 1-like protein	0.18684	-2.4201	6.57E-05	4.1823	0.862434	2.06E-05	-1.84198	2
163	P01023	Alpha-2-macroglobulin	2.0399	1.0285	8.98E-05	4.0468	0.899471	3.00E-05	1.606191	4
668	Q9BQ50	Three prime repair exonuclease 2	0.49069	-1.0271	8.98E-05	4.0468	0.915344	3.01E-05	-0.59433	5
270	P08603	Complement factor H	2.6697	1.4167	0.000116	3.9364	0.878307	3.97E-05	2.119093	1
893	P02647	Apolipoprotein A-I	0.37301	-1.4227	0.000131	3.884	0.910053	4.68E-05	-1.06923	5
714	Q96QP1	Alpha-protein kinase 1	0.28642	-1.8038	0.00014	3.8545	0.931217	5.22E-05	-1.19482	5
763	P62906	60S ribosomal protein L10a	0.2856	-1.8079	0.00021	3.6776	0.89418	9.09E-05	-1.44914	2
695	P01009	Alpha-1-antitrypsin	2.7741	1.472	0.00021	3.6768	0.904762	9.27E-05	1.689427	3
896	P02647	Apolipoprotein A-I	0.4723	-1.0822	0.000229	3.6408	0.957672	0.000104	-0.79204	5
567	Q96PX9	Pleckstrin	0.42457	-1.2359	0.000249	3.6045	0.873016	0.000115	-0.74687	5
291	P01023	Alpha-2-macroglobulin	2.0217	1.0156	0.000266	3.5757	0.835979	0.000125	1.632464	1
591	O00487	26S proteasome non-ATPase regulatory subunit 14	0.32219	-1.634	0.000278	3.5558	0.899471	0.000135	-1.42281	5
689	P01009	Alpha-1-antitrypsin	3.4951	1.8053	0.000283	3.5489	0.941799	0.000141	1.883603	3
589	P02768	Serum albumin	0.40156	-1.3163	0.000283	3.5489	0.883598	0.000141	-0.99896	5
1012	Q6ZN57	Zinc finger protein 2 homolog, Zfp-2	0.32887	-1.6044	0.000308	3.5114	0.899471	0.000156	-1.20552	5

570	P02768	Serum albumin	0.36477	-1.455	0.000337	3.4728	0.878307	0.000173	-1.0454	5
900	P02647	Apolipoprotein A-I	0.37379	-1.4197	0.000404	3.3931	0.94709	0.000211	-1.2312	5
782	P00738	Haptoglobin	0.28009	-1.836	0.000458	3.3389	0.883598	0.000243	-1.47889	2
771	P01024	Complement C3	2.1743	1.1206	0.001114	2.953	0.883598	0.000657	1.529116	3
688	P01009	Alpha-1-antitrypsin	2.66	1.4114	0.001286	2.8907	0.862434	0.000778	1.632814	3
820	P02647	Apolipoprotein A-I	0.38951	-1.3603	0.001719	2.7648	0.883598	0.001052	-0.88809	2
710	P01008	Antithrombin-III	0.4056	-1.3019	0.002893	2.5386	0.846561	0.001943	-0.93678	5
966	P00738	Haptoglobin	0.2724	-1.8762	0.008617	2.0647	0.793651	0.006109	-1.62137	2
759	P00738	Haptoglobin	0.48635	-1.0399	0.009354	2.029	0.84127	0.006701	-0.85225	2
596	P00450	Ceruloplasmin	2.1998	1.1374	0.013083	1.8833	0.772487	0.009764	1.475433	3
982	Q8TAA9	Vang-like protein 1	0.4021	-1.3144	0.02015	1.6957	0.783069	0.015489	-1.53285	2
766	Q6UXP9	Putative uncharacterized protein	2.1863	1.1285	0.035418	1.4508	0.746032	0.029339	1.197646	3
972	Q9NR11	Zinc finger protein 302	2.9438	1.5577	0.039396	1.4045	0.756614	0.033594	1.735095	3

Table S4. Experimental design: 28 patient samples and 10 controls run on 19 2D-PAGE gels, samples were labeled randomly with Cy3 and Cy5, and a pooled sample was used as an internal standard and was stained with Cy2.

Gel	Cy3	Cy5	Cy2
1	CF1(III)	CF15 (IV)	Pooled sample
2	CF16 (IV)	CF2 (III)	Pooled sample
3	CF3(III)	CF17 (IV)	Pooled sample
4	C1	CF4 (III)	Pooled sample
5	CF5 (III)	C2	Pooled sample
6	C4	CF6 (III)	Pooled sample
7	CF18 (IV)	C5	Pooled sample
8	C6	CF19 (IV)	Pooled sample
9	CF7 (III)	CF20 (IV)	Pooled sample
10	CF21 (IV)	CF8 (III)	Pooled sample
11	CF9 (III)	CF22 (IV)	Pooled sample
12	23 (IV)	C7	Pooled sample
13	C8	CF10 (III)	Pooled sample
14	CF24 (IV)	C9	Pooled sample
15	C10	CF25 (IV)	Pooled sample
16	26 (IV)	CF11 (III)	Pooled sample
17	CF12 (III)	CF27 (IV)	Pooled sample
18	CF28 (IV)	CF13 (III)	Pooled sample
19	CF14 (III)		Pooled sample

Table S5. Experimental conditions of the selected proteins' signature peptides used for proteomics validation.

Accession Number	Protein Name	Peptide Sequence [location]	Length (AA)	Alignment score BLAST/NCBI	Molecular weight (g/mol)	MRM Transition		Cone voltage (v)	Collision energy (v)	RT (min)
						MS1 (m/z)	MS2 (m/z)			
P02647 (D)	Apolipoprotein A-I (APOA1)	R.EQLGPVTQEFWDNLEK.E [85, 100]	16	50-80	1933.2	967.67	84.08	100	98	1.75
P01024 (U)	Complement C3 (C3)	R.SNLDEDIIAEENIVSR.S [748, 763]	16	50-80	1816.9	909.7	86.16	48	104	1.63
P05156 (D)	Complement factor I (C3b/C4b)	R.VFSLQWGEVK.L [480, 489]	10	<40	1192.3	597.0	120.07	38	40	1.73
P00738 (D)	Haptoglobin (Hp)	R.TEGDGVYTLNNEK.Q [118, 130]	13	40-50	1439.5	720.58	84.15	44	78	1.26
P02753 (D)	Retinol-binding protein 4 (RBP4)	K.YWGVASFLQK.G [107, 116]	10	<40	1198.4	600.22	136.11	36	56	1.76
P00450 (U)	Ceruloplasmin (CP)	K.NNEGTYYSPNYNPQSR.S [484, 499]	16	40-50	1903.2	635.67	70.12	28	52	1.27
